# Norovirus and Foodborne Disease, United States, 1991–2000

**DOI:** 10.3201/eid1101.040426

**Published:** 2005-01

**Authors:** Marc-Alain Widdowson, Alana Sulka, Sandra N. Bulens, R. Suzanne Beard, Sandra S. Chaves, Roberta Hammond, Ellen D.P. Salehi, Ellen Swanson, Jessica Totaro, Ray Woron, Paul S. Mead, Joseph S. Bresee, Stephan S. Monroe, Roger I. Glass

**Affiliations:** * Centers for Disease Control and Prevention, Atlanta, Georgia, USA; † Atlanta Research and Education Foundation, Atlanta, Georgia, USA; ‡ Department of Human Resources, Atlanta, Georgia, USA; § Bureau of Community Environmental Health, Tallahassee, Florida, USA; ¶ Ohio Department of Health, Columbus, Ohio, USA; # Department of Health, Minneapolis, Minnesota, USA; ** Maryland Department of Health and Mental Hygiene, Baltimore, Maryland, USA; †† New York State Department of Health, Troy, New York, USA

**Keywords:** research, food, norovirus, disease outbreaks, burden of illness

## Abstract

Efforts to prevent foodborne illness target bacterial pathogens, yet noroviruses (NoV) are suspected to be the most common cause of gastroenteritis. New molecular assays allow for better estimation of the role of NoV in foodborne illness. We analyzed 8,271 foodborne outbreaks reported to the Centers for Disease Control and Prevention from 1991 to 2000 and additional data from 6 states. The proportion of NoV-confirmed outbreaks increased from 1% in 1991 to 12% in 2000. However, from 1998 to 2000, 76% of NoV outbreaks were reported by only 11 states. In 2000, an estimated 50% of foodborne outbreaks in 6 states were attributable to NoV. NoV outbreaks were larger than bacterial outbreaks (median persons affected: 25 versus 15), and 10% of affected persons sought medical care; 1% were hospitalized. More widespread use of molecular assays will permit better estimates of the role of NoV illness and help direct efforts to control foodborne illness.

## Abstract 2

Analysis of foodborne outbreaks shows how advances in viral diagnostics are clarifying the causes of foodborne outbreaks and determining the high impact of norovirus infections.

Foodborne infections are estimated to cause 76 million illnesses, 300,000 hospitalizations, and 5,000 deaths annually in the United States (*1*). Several high-profile outbreaks in the last 15 years have focused attention on the role of bacteria in severe foodborne illness (*2*–*4*) and led to serious efforts to prevent bacterial contamination of food during all levels of processing and handling—the “farm-to-fork” model. However, in more than two thirds of outbreaks of foodborne illness, no pathogen is identified (*5*).

Noroviruses (NoV), previously known as Norwalk-like viruses, have long been suspected to be a frequent cause of foodborne outbreaks (*6*–*11*). Until recently, diagnosis of NoV infection relied on methods that were insensitive (electron microscopy [*12*]), difficult to set up (serologic testing with human reagents [*13*]), and available only in research settings. In 1982, epidemiologic and clinical criteria were formulated to help attribute outbreaks to NoV in the absence of a simple diagnostic test (*14*). Despite these criteria, the absence of any routine diagnostic assay for NoV has discouraged investigations into outbreaks of suspected viral etiology and thus limited assessment of the true impact of gastroenteritis associated with these pathogens. In 2000, for example, a survey of public health professionals in Tennessee found that only 9% cited viruses as a major cause of foodborne illness (*15*). Not unexpectedly, therefore, of the 2,751 foodborne outbreaks reported to the Centers for Disease Control and Prevention (CDC) from 1993 to 1997, only 9 (0.3%) were confirmed as due to NoV (*5*).^1^

In the early 1990s, sensitive and simpler assays were developed to detect NoV by identifying viral RNA after reverse transcription-polymerase chain reaction (RT-PCR) (*16*). In 1993, RT-PCR was adopted at CDC for the routine detection of NoV (*17*), particularly in outbreaks in which specimens test negative for common bacteria. A number of state public health laboratories subsequently adopted similar assays or began sending specimens to CDC for NoV testing. When RT-PCR was used, a NoV was identified as the etiologic agent in 93% of outbreaks of nonbacterial gastroenteritis submitted for testing to CDC from 1997 to 2000 (*18*). However, this selection was of specimens from outbreaks of illness characteristic of viral infection, and they usually have already tested negative for bacteria. The selection introduces bias since it does not permit an assessment of the true relative frequency of foodborne outbreaks of NoV disease. Therefore, we analyzed data from all foodborne outbreaks (irrespective of cause) reported to CDC by state health departments from 1991 through 2000 to assess how recent application of RT-PCR techniques might have improved understanding of the relative impact and role of NoV in these outbreaks in the United States.

## Methods

We used 3 related datasets: 1) all foodborne outbreaks reported to CDC from 1991 through 2000 (N = 8,271), 2) a subset of these outbreaks reported from 1998 though 2000 when surveillance was enhanced and states began to use NoV diagnostics (N = 4,072), and 3) all foodborne outbreaks reported in 2000 in 6 selected states from which supplementary data on diagnostic testing were gathered (N = 600).

### Foodborne Outbreak Reports, 1991–2000

Outbreaks of foodborne disease (excluding those on cruise ships) are voluntarily reported by state health departments to CDC for inclusion in the National Foodborne Outbreak Reporting System. Whether an outbreak is classified as foodborne or not is at the discretion of the state epidemiologist. Minimum data required for registering an outbreak report include the number of persons ill and the date of onset of the first case. The determination of outbreak cause is based on CDC’s pathogen-specific guidelines (*19*). In 1998, the surveillance system was enhanced by annual data verification with states and solicitation of any unreported outbreaks.

We reviewed records of 8,271 foodborne outbreaks reported to CDC from 1991 through 2000. We also noted the year in which state laboratories set up the RT-PCR assay for NoV, and by cross-referencing with CDC laboratory logs, we determined whether an outbreak had been confirmed as attributable to NoV at a laboratory in a state or at CDC.

### Foodborne Outbreak Reports, 1998–2000

This subset of foodborne outbreaks was selected for further analysis because, in addition to enhanced surveillance in this period, state public health laboratories had begun to test routinely for NoV, and these reports therefore included most outbreaks of confirmed NoV disease. Available variables included the laboratory-confirmed cause; clinical data (symptoms, median incubation period, median duration of illness); food vehicle; whether a foodhandler was implicated; and the number of persons exposed, ill, requiring medical attention, or hospitalized.

From January 1998 through December 2000, a total of 4,072 outbreaks were reported to CDC. We excluded 30 outbreaks involving multiple states and 10 occurring in the U.S. territories and further analyzed the remaining 4,032 outbreak reports.

To assess the differences between states in outbreak reporting and laboratory testing, each state was classified into 1 of 5 groups on the basis of the number of NoV-confirmed outbreaks that a state reported in 1998 to 2000 (>20, 10–19, 5–9, 1–4, or none reported). The proportion of reported outbreaks with a known cause and the proportion confirmed to be due to NoV were calculated for each group. The number of reported outbreaks per 100,000 population per state for these 3 years was also calculated by using U.S. Census data for 2000.

To characterize the severity of illness and the settings associated with NoV outbreaks, we selected the 305 NoV-confirmed outbreaks and analyzed those with complete information on medical care (n = 112) and setting (n = 278). We calculated the proportion of persons seeking care and the proportion hospitalized by using the number of case-patients interviewed as a denominator.

To compare the epidemiologic and clinical features of outbreaks attributed to bacteria and viruses, we selected, from the 4,032 outbreaks of gastroenteritis, a subset of 1,216 reports with complete information on the number ill, duration of illness, incubation period, and the proportion of interviewed patients who reported vomiting or fever. Of these outbreaks, 136 were attributed to NoV, 173 to bacteria, and 907 to an undetermined cause. We further compared outbreak reports with information on implicated food types (n = 608) and whether or not an ill foodhandler was thought involved by the outbreak investigators (n = 760).

### Data on Specimen Screening from 6 States, 2000

Data on the pathogens screened in a single outbreak are not reported to CDC; therefore, to estimate the proportion of outbreaks that would be NoV-confirmed if collected specimens were tested routinely not only for bacteria but also for NoV, we gathered additional data on the testing of stools gathered from foodborne outbreaks in 2000 from 6 states (Georgia, Minnesota, Ohio, Florida, Maryland, New York). These states were selected because they collected stools from a large number of outbreaks and had laboratory capability to test specimens for NoV.

We applied the proportion of all outbreaks tested for NoV that were NoV-positive in each state (>1 positive specimens) to the number of outbreaks of undetermined etiology for which specimens had been gathered, had tested negative for bacteria, but had not been tested for NoV. We then added this figure to the total actual number of NoV outbreaks to estimate the proportion of all outbreaks with specimens in that state that would be attributable to NoV had specimens from all outbreaks been tested fully.

## Results

### Foodborne Outbreak Reports, 1991–2000

**Figure 1:**
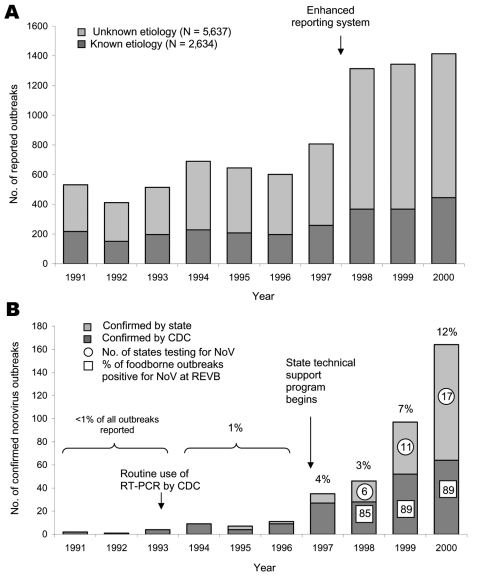
A) Foodborne outbreaks reported to the Centers of Disease Control and Prevention (CDC), United States, 1991–2000. B) Norovirus (NoV)-confirmed foodborne outbreaks reported to CDC, United States, 1991–2000. REVB, Respiratory and Enteric Branch, CDC; RT-PCR, reverse transcription–polymerase chain reaction. Percentage value above bars represents proportion of all foodborne outbreaks reported to CDC that were laboratory-confirmed to be due to NoV by REVB and by some state public health laboratories.

The number of foodborne outbreaks reported to CDC per year from 1991 to 2000 ranged from 411 outbreaks in 1992 to 1,414 in 2000, and increased markedly in 1998, when the reporting system was changed (Figure 1). Of 8,271 outbreaks, 5,637 (68%) were of undetermined etiology. The number of NoV-confirmed outbreaks increased markedly from 11 outbreaks in 1996 to 164 (12% of all reported outbreaks) in 2000. This rise was initially due to laboratory confirmation of NoV by CDC, but by 2000, 100 (61%) of 164 NoV outbreaks were confirmed in state laboratories. Underreporting, however, remained an obvious problem since only 17 (34%) of 50 state public health laboratories tested for NoV, while the remaining 33 states (66%) either sent specimens to CDC for diagnosis (n = 12), or did not report any NoV outbreaks (n = 21).

### Foodborne Outbreak Reports, 1998–2000

Of 4,032 outbreaks reported in this period of enhanced surveillance, only 1,146 (28%) were of determined cause and 2,886 (72%) were of undetermined etiology (Table 1). NoV-confirmed outbreaks comprised 305 (8%) of all 4,032 outbreaks or 27% of the 1,146 outbreaks with a determined cause. These 305 NoV outbreaks accounted for 13,527 (18%) of all 74,481 sick persons in all 4,032 outbreaks or 39% of 34,539 sick persons in 1,146 outbreaks of known cause.

**Table 1:** Foodborne outbreaks of gastroenteritis of known and unknown etiology by states grouped by number of reports of norovirus (NoV)-confirmed outbreaks, United States, 1998–2000

No. of NoV outbreaks reported by states	No. of states reporting	All reported outbreaks			NoV outbreaks reported	
		Total no. (R*)	Determined etiology (%)	Undetermined etiology (%)	No. (% of all outbreaks)	% of all outbreaks with determined etiology
>20	2	382 (2.3)	166 (43)	216 (57)	94 (25)	57
11–20	9	2,273 (2.3)	447 (20)	1,826 (80)	138 (6)	31
6–10	4	304 (0.8)	136 (45)	168 (55)	33 (11)	24
<5	21	830 (0.9)	269 (32)	561 (68)	40 (5)	15
None	15†	243 (0.8)	128 (53)	115 (47)	0 (0)	0
Total	51	4,032 (1.4)	1,146 (28)	2,886 (72)	305 (8)	27

*R = outbreaks reported per 100,000 population, using U.S. Census data 2000. †Includes District of Columbia.

### NoV Reporting

**Figure 2:**
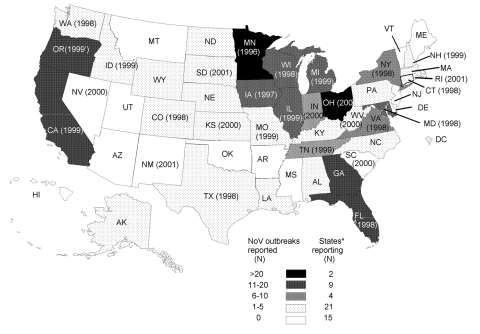
Norovirus-confirmed foodborne outbreaks by state, United States, 1998–2000 (N = 305).Years in parenthesis indicate first year a state public health laboratory developed molecular assays for norovirus (as of December 2001). *Includes District of Columbia.

A great disparity was observed in the reporting of NoV outbreaks. Of the 50 U.S. states and the District of Columbia, 15 (29%) reported no NoV outbreaks (Figure 2). Of the total of 305 NoV outbreaks, 232 (76%) were reported by 11 states, which each investigated >10 NoV outbreaks and accounted for 613 (53%) of all 1,146 outbreaks of determined cause.

We hypothesized that the proportion of outbreaks of determined cause reported in each state would be lowest in those states not reporting any NoV-confirmed outbreaks, but this hypothesis was not supported by the data. In fact, paradoxically, the 15 states that reported no NoV outbreaks in the study period determined a cause in 53% of all outbreaks, compared to 20%–45% in the 35 states that reported at least 1 NoV outbreak. The 11 states that reported >10 NoV outbreaks also reported, on average, more outbreaks per 100,000 population (2.3) compared with the 35 states that reported 0–10 NoV outbreaks (0.8–0.9). The number of NoV outbreaks reported by states, however, was not simply a function of total outbreaks reported; the percentage of NoV outbreaks of those outbreaks of determined etiology also increased significantly, from 0% to 57% (chi square for trend; p > 0.001), which suggests better outbreak investigation and testing for NoV.

### Illness

Information on physician visits and hospitalization was complete in 112 (37%) of all 305 NoV outbreaks. Of 3,370 persons affected in these 112 outbreaks, 329 (10%) sought care from a physician, and 33 (1%) were hospitalized.

### Setting

For 278 (91%) of the 305 NoV outbreaks where the site of food consumption or preparation was recorded, restaurants, caterers, or food outlets were associated with 108 (39%), private homes with 35 (13%), daycare facilities or schools with 27 (10%), workplace with 18 (6%), nursing homes or hospitals with 14 (5%), and other settings with 76 (27%).

### Comparison of Bacterial and NoV Outbreaks

We compared selected epidemiologic and clinical features of NoV outbreaks (n = 136), bacterial outbreaks (n = 173), and outbreaks of unknown etiology (n = 907), when information was complete. Of the 173 bacterial outbreaks, 79 (46%) were attributed to *Salmonella* spp., 27 (16%) to *Clostridium* spp., 20 (12%) to *Staphylococcus aureus,* 19 (11%) to *Shigella* spp., 13 (8%) to *Escherichia coli*, 7 (4%) to *Bacillus cereus*, 6 (3%) to *Campylobacter* spp., and 2 (1%) to other bacterial pathogens.

NoV outbreaks were significantly larger than outbreaks of bacterial or unknown etiology (median number of cases per outbreak = 25 versus 15 and 7, respectively. Wilcoxon rank sum test: p < 0.001) (Table 2). Viral outbreaks had a shorter duration of illness compared to bacterial outbreaks but one similar to that of outbreaks of unknown etiology (median duration <48 hours = 82%, 40%, and 85%, respectively). Vomiting was more often a predominant symptom (reported by >50% of ill persons) in NoV outbreaks than in outbreaks of bacterial or unknown etiology (p = 0.001) and was reported in all 136 NoV outbreaks. Fever, however, was less often reported in outbreaks of NoV disease.

**Table 2:** Selected epidemiologic and clinical features of foodborne outbreaks of gastroenteritis of noroviral, bacterial, and unknown cause, United States, 1998–2000*

Features†	Etiology of outbreak			p value‡
	Norovirus (N = 136) (%)	Bacterial (N = 173)(%)	Unknown (N = 907) (%)	
No. of persons ill				
<10	22 (16)	65 (38)	544 (60)	< 0.001
>10	114 (84)	108 (62)	363 (40)	
Median no. of persons/outbreak (range)	25 persons (2–199)	15 persons (2–736)	7 persons (2–800)	0.001§
Median duration of illness (h)				
<48	111 (82)	70 (40)	763 (85)	< 0.001¶
>48	25 (18)	103 (60)	134 (15)	
Median incubation period (h)				
<24	21 (15)	105 (61)	517 (57)	< 0.001
25–48	82 (60)	13 (7)	266 (29)	
>48	33 (25)	55 (32)	124 (14)	
% of persons vomiting				
<50	19 (14)	114 (68)	352 (39)	< 0.001
>50	117 (86)	59 (32)	555 (60)	
% of persons with fever				
<50	90 (66)	100 (57)	752 (83)	< 0.001
>50	46 (34)	73 (42)	155 (17)	

*Data are no. (%), unless otherwise noted. †No significant differences found in proportions of ill persons with diarrhea or abdominal cramping. ‡Chi-square test for unequal odds unless otherwise noted. p value refers to comparison of norovirus (NoV) outbreaks to both bacterial and unknown outbreaks separately unless otherwise noted. §Wilcoxon rank sum test comparing median values. ¶Significant association only between NoV and bacterial outbreaks.

The median incubation period was significantly longer in outbreaks of NoV gastroenteritis: 85% of these outbreaks featured a median incubation period >24 hours compared with 39% in outbreaks of bacterial cause and 43% in outbreaks of unknown etiology. This finding is largely explained by outbreaks caused by preformed toxins from certain bacteria (*S. aureus*, *Clostridium perfringens*, *B. cereus*), which tend to have shorter incubation periods.

NoV outbreaks were strongly associated with eating salads, sandwiches, and produce: these items were implicated in 56% of the 76 NoV outbreaks in which a food item was identified, compared with 19% of 124 bacterial outbreaks and 28% of 408 outbreaks of unknown etiology (chi-square test: p < 0.05) (Table 3). NoV outbreaks were significantly less often associated with meat dishes than bacterial outbreaks and outbreaks of unknown etiology (11% versus 44% and 34%, respectively: p < 0.05). A foodhandler was more likely to be implicated in a NoV outbreak (48% of 94 outbreaks with available data) than in either a bacterial outbreak (20% of 102 outbreaks) or an outbreak of unknown etiology (9% of 564 outbreaks) (p < 0.001).

**Table 3:** Role of different foods and foodhandlers in outbreaks of gastroenteritis of noroviral, bacterial, and unknown cause, United States, 1998–2000*

	Cause of outbreak			p value†
	Norovirus no. (%)	Bacteria no. (%)	Unknown no. (%)	
Total outbreaks with data on implicated food	76	124	408	
Implicated food				
Salad	20 (26)	20 (16)	73 (18)	NS
Sandwich	10 (13)	0	24 (6)	< 0.05‡
Produce/fruit	13 (17)	4 (3)	15 (4)	< 0.001
Meat dish	8 (11)	50 (40)	139 (34)	< 0.001
Fish dish	4 (5)	9 (7)	19 (5)	NS
Bakery product	5 (7)	2 (2)	15 (4)	NS
Oysters	2 (3)	2 (2)	12 (3)	NS
Other various§	14 (18)	37 (30)	111 (27)	ND
Total outbreaks with data on investigation of foodhandler	94	102	564	
Foodhandler implicated				
Yes	45 (48)	20 (20)	51 (9)	< 0.001
No	49 (52)	82 (80)	513 (91)	

* ND, not done; NS, not significant. †Chi-square test: p value refers to comparison of norovirus (NoV) outbreaks with both bacteria and unknown outbreaks separately. ‡p < 0.001 when comparing NoV outbreaks with bacterial outbreaks. §No difference noted for dairy products (3%–5%), cold meats (2%–4%), egg dishes (0%–1%), beverages (1%–4%), or ice (0%–1%).

### Specimen Screening Data from 6 States, 2000

In the 6 states for which data on specimen testing were obtained, the percentage of outbreaks tested for NoV that were positive was 44%–100%, and the total percentage in all 6 states was 79% (Table 4). Even in these states, NoV testing was much less likely to be performed than was testing for bacteria. Of 220 outbreaks from which stool samples were collected, specimens from 85 (39%) were tested for NoV compared to 207 (94%) tested for bacteria. Specimens from 55 outbreaks (25%) tested negative for bacteria, but no further testing for viruses was performed. The overall percentage of all outbreaks with specimens that tested positive for NoV was 30%, but in 2 states that tested all specimens for NoV (Georgia and Minnesota), the average percentage was 43% (22/51) compared with 27% (45/169) in the 4 other states that did not test fully for NoV. Assuming that these 4 states had tested specimens from these outbreaks for NoV, 110 (50%) of the 220 outbreaks with specimens collected in all 6 states would have been confirmed as caused by NoV.

**Table 4:** Laboratory testing of fecal specimens from foodborne outbreaks of gastroenteritis and projected number of norovirus (NoV)-confirmed outbreaks in 6 states, 2000

State	Total reported outbreaks	Total with specimens (% of total outbreaks)	No. positive/no. tested						No. with unknown etiology not tested for NoV*	Total NoV outbreaks (% outbreaks with specimens)	
			Tested only for bacteria	Tested only for NoV	Tested for both bacteria and NoV		Total for bacteria (%)	Total for NoV (%)			
					Bacteria	NoV				Actual	Projected†
MD	116	42 (36)	13/35	2/2	0/5	5/5	13/40 (33)	7/7 (100)	22	7 (17)	29 (69)
MN	41	32 (78)	10/10	0/1	2/21	15/21	12/31 (39)	15/22 (68)	0	15 (47)	15 (47)
GA	26	19 (73)	9/9	0/0	2/10	7/10	11/19 (58)	7/10 (70)	0	7 (37)	7 (37)
NY	60	35 (58)	19/28	1/2	0/5	4/5	19/33 (58)	5/7 (71)	9	5 (14)	11 (31)
FL	274	40 (15)	11/26	1/3	0/6	3/6	11/32 (47)	4/9 (44)	11‡	4 (10)	9 (23)
OH	83	52(63)	8/22	0/0	0/30	29/30	8/52 (15)	29/30(97)	13§	29 (56)	42 (81)
Total	600	220 (37)	70/130	4/8	4/77	63/77	74/207 (38)	67/85 (79)	55	67 (30)	110 (50)^b^

*Derived by subtracting nominator from denominator in column 4, i.e., the number of outbreaks tested only for bacteria and with negative test results. †Calculated by multiplying value in column 10 by percentage in column 9 and adding to value in column 11. ‡Excludes 4 confirmed outbreaks of other causes (3 of *Cryptosporidium* and 1 of chemical cause). ^§^Excludes 1 confirmed outbreak of chemical cause.

## Discussion

The introduction of RT-PCR in the 1990s increased the percentage of all outbreaks attributable to NoV in the Unites States from <1% in 1991 to 12% in 2000. Nonetheless, noroviruses remain grossly underestimated as a cause of gastroenteritis outbreaks. From 1998 through 2000, most NoV outbreaks (76%) were reported from 11 states; 36 states, generally those with no PCR capability, reported either few or no outbreaks. Using data from 6 states, we estimated that if all specimens were tested for viruses, half of all foodborne outbreaks in the United States could be attributable to NoV. Even in these 6 states, bacteria were more likely to be tested for than viruses; specimens from 25% of outbreaks were negative for bacteria but not further tested. We also show that NoV outbreaks affect almost 50% more persons than in bacterial outbreaks (median = 25 versus 15 persons affected). Although NoV outbreaks were generally of short duration, symptoms were sufficiently severe in 9.8% of patients to require medical care and in 1%, hospitalization.

In addition to a historic lack of diagnostic assays, a further reason for underrecognition of NoV is a lack of specimens and epidemiologic information gathered from outbreaks that exhibit clinical features characteristic of viral gastroenteritis. We expected states that do not test for NoV to report more outbreaks of unknown etiology, but this was not the case. In fact, states that reported no NoV outbreaks also reported the lowest percentage of outbreaks with an undetermined etiology (47%, Table 1). This bias in the etiologic distribution of reported outbreaks toward bacterial causes that can be easily determined is further suggested by the lower number of outbreak reports in states with <10 NoV outbreaks from 1998 though 2000 (0.8–0.9 outbreaks/100,000 persons) compared with those states that reported >10 NoV outbreaks (2.3 outbreaks/100,000 persons). Genuine differences in the incidence of NoV disease (e.g., rural/urban) or different patterns of reporting disease among communities in different states are also possible.

We found that >56% of foodborne NoV outbreaks were associated with eating salads, sandwiches, or fresh produce, which confirms that contamination of foods requiring handling but no subsequent heating is an important source of NoV infection (*9**,**20**–**22*). Despite their well-documented role in large multistate NoV outbreaks (*23**–**25*), oysters have not been frequently associated with NoV disease in the last 10 years in the United States. We excluded only 2 multistate NoV outbreaks from the analysis, 1 of which was linked to oysters. Restaurants or caterers were associated with 39% of NoV outbreaks, yet in >50% of NoV outbreaks, no foodhandler was implicated. This finding probably reflects a lack of positive evidence rather than the actual ruling out of a foodhandler’s involvement. Although asymptomatic infections may play a role in transmission (*26**,**27*), and foodhandlers are likely to underreport illness, some outbreaks with no foodhandler implicated may be due to contamination of fresh produce at the source, as has been previously documented for NoV (*21**,**27*) and other foodborne viruses transmitted by the feco-oral route (*28*).

Our projected number of NoV outbreaks in each state may be overestimated because outbreaks that were tested for NoV were likely to have been more characteristic of NoV disease than those not tested. However, we only applied the proportion of outbreaks positive for NoV (79%) to outbreaks of unknown etiology that had already tested negative for bacteria. Moreover, between them, the 2 states that tested all nonbacterial outbreaks for NoV found 43% of outbreaks attributable to NoV, consistent with our estimate from all 6 states. Biases in surveillance data complicate straightforward extrapolation of our estimate of outbreaks with specimens from 6 states, to the group of reported outbreaks with no specimens collected in the same 6 states and in other states. Certain clinical characteristics of outbreaks of unknown etiology were similar to those of NoV outbreaks (e.g., percentage of patients vomiting); other epidemiologic characteristics were similar to those for bacterial outbreaks (e.g., implicated food). Etiologic make-up of outbreaks with no specimens collected is also likely to differ between states. Since specimens remain less likely to be collected from outbreaks of acute gastroenteritis of short duration, we think our estimate can be reasonably extrapolated to all outbreaks of unknown etiology.

Only a few small studies have looked at the relative impact of NoV as a cause of foodborne illness (Table 5), and none have fully tested for NoV with PCR. A small study of enhanced surveillance during 1 year in a Swedish municipality found 6% of all foodborne outbreaks, but 38% of 13 that were laboratory-confirmed, to be attributable to caliciviruses (*30*). Our estimate of 50% of foodborne outbreaks being attributable to NoV is higher than estimates that rely on epidemiologic criteria (33%–41%) (*6*,*8*), consistent with the low sensitivity of such criteria (CDC, unpub. data). Our estimate of percentage of outbreaks attributable to NoV is lower than Mead’s figure of 66% of all foodborne illness of known etiology being caused by NoV (*1*). However, our finding that NoV outbreaks are >50% larger than bacterial outbreaks suggests that the total number of cases associated with our estimate of outbreaks is comparable to Mead’s estimate. We may have overestimated the size of NoV outbreaks and the proportion of persons seeking care since these larger outbreaks of more serious illness may be more likely to be reported. However, our estimates are not inconsistent with a study in the United Kingdom that reported the median size of NoV outbreaks to be 21 persons and the hospitalization rate to be 0.3% (*32*). The very low infective dose of NoV (*33*) allows for extensive transmission by means of contaminated food and subsequent person-to-person spread. Data on other variables may also be biased. For instance, that 61% of bacterial outbreaks would have a median incubation of <24 hours is surprising, given that 69% of the analyzed bacterial outbreaks were attributed to *Salmonella* spp., *Shigella* spp., *Campylobacter* *spp*., and *E. coli*, which have longer incubation periods. Finally, since no standard criteria are required for an outbreak to be classified as foodborne and since NoV are more often spread from person-to-person than bacteria, the dataset from 6 states that we used may have resulted in an overestimate of the impact of foodborne NoV.

**Table 5:** Estimates of the role of norovirus (NoV) in foodborne outbreaks of gastroenteritis*

Place (reference)	Years of data	No. of foodborne outbreaks	Method used to attribute to NoV	% of foodborne outbreaks attributable to NoV
United Kingdom (*31*)	1995–1996	341	Electron microscopy	6
Sweden (*30*)	1998–1999	85	RT-PCR	6
Sweden (*29*)	1994–1998	92	Electron microscopy	72
New Zealand†	2000–2002	383	RT-PCR	12
The Netherlands‡	2002	59	RT-PCR	27
United States (*6*)	1982–1989	1049	Epidemiologic criteria	33
United States (*8*)	1981–1998	295	RT-PCR and epidemiologic criteria	41
United States§	2000	600	RT-PCR and extrapolation	50

*RT-PCR; reverse transcription–polymerase chain reaction. †N. Boxall, pers. comm. ‡Y. van Duynhoven, pers. comm. §Current study.

Efforts are required to increase the capacity of states to investigate outbreaks, irrespective of suspected cause, and include improved specimen collection and more widespread testing for viruses. Evaluation of epidemiologic criteria is needed to assess how best these can be used to guide testing strategies when laboratory resources are limited. Better appreciation of the exact causes of the large number of outbreaks of undetermined etiology will help better target measures to prevent foodborne disease. Furthermore, to be able to identify novel and intentionally introduced pathogens, the ability of state health departments to quickly investigate outbreaks and discount common causes is critical. “Real-time” collection systems of epidemiologic and sequence data from different outbreaks, such as developed in Europe (*34*), can provide insights into the epidemiology of NoV (*35*) and will allow for rapid comparison of data to rapidly identify common risk factors (such as foods contaminated at source) and implement control measures. While these initiatives are developed, however, the high disease impact of outbreaks of NoV illness should prompt prioritization of development and implementation of prevention measures, such as foodhandler education, by food safety agendas.

## Footnotes

1 Efforts in 1998 to improve outbreak reporting resulted in more outbreaks being retrospectively attributed to this period. The current figures for 1993 to 1997 are 65 (2%) of 3,257 outbreaks attributable to NoV and 67% of unknown etiology.
